# Mitochondria‐Associated Membranes: A Key Point of Neurodegenerative Diseases

**DOI:** 10.1111/cns.70378

**Published:** 2025-05-23

**Authors:** Yiwei Zhang, Xiuqin Rao, Jiayi Wang, Hantian Liu, Qixian Wang, Xifeng Wang, Fuzhou Hua, Xilong Guan, Yue Lin

**Affiliations:** ^1^ Department of Anesthesiology, The Second Affiliated Hospital, Jiangxi Medical College Nanchang University Nanchang Jiangxi Province China; ^2^ Jiangxi Provincial Key Laboratory of Anesthesiology Nanchang Jiangxi Province China; ^3^ Queen Mary College Nanchang University Nanchang Jiangxi Province China; ^4^ Department of Anesthesiology, The First Affiliated Hospital, Jiangxi Medical College Nanchang University Nanchang Jiangxi Province China; ^5^ Department of Anesthesiology Yingtan City People's Hospital Yingtan City Jiangxi Province China

**Keywords:** Alzheimers' disease, Ca^2+^ homeostasis, IP3R, mitochondria‐associated membranes, neurodegenerative disease, Parkinson's disease, Sig‐1R, VAPB

## Abstract

**Background:**

Neurodegenerative diseases pose significant health challenges in the 21st century, with increasing morbidity and mortality, particularly among the elderly population. One of the key factors contributing to the pathogenesis of these diseases is the disrupted crosstalk between mitochondria and the endoplasmic reticulum. Mitochondria‐associated membranes (MAMs), which are regions where the ER interfaces with mitochondria, serve as crucial platforms facilitating communication between these organelles.

**Objectives:**

This review focuses on the structural composition and functions of MAMs and highlights their roles. Additionally, in this review, we summarize the relationship between MAM dysfunction and various neurodegenerative diseases, including Alzheimer's disease, Parkinson's disease, and others. The involvement of key proteins such as Sig‐1R, IP3R, and VAPB in maintaining ER‐mitochondrial communication and their dysfunction in neurodegenerative diseases is emphasized.

**Conclusion:**

Through analyzing the effects of MAM on neurodegenerative diseases, we provide the newest insights and potential therapeutic targets for the treatment of these debilitating conditions.

## Graphical abstract

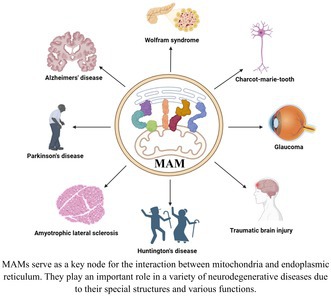

**Abbreviations**
ACAT: acyl coenzyme A: cholesterol acyltransferase
AD: Alzheimer's disease
ALS: amyotrophic lateral sclerosis
Apo‐E: apolipoprotein E
APP: amyloid precursor protein
ASC: apoptosis‐associated speck‐like protein containing a CARD
ATAD3A: AAA ATPase domain‐containing protein 3A
ATF6: activating transcription factor 6
ATG14: autophagy‐related gene 14
ATP: adenosine triphosphate
AUTOTAC: autophagy‐targeting chimera
Aβ: β‐amyloid
BACE1: β‐site amyloid precursor protein cleaving enzyme 1
BAP31: B‐cell receptor associated protein 31
BECN1: recombinant beclin 1
BiP: binding immunoglobulin protein
CAV1: caveolin‐1
CDS2: CDP‐diacylglycerol synthase‐2
CHOP: C/EBP homologous protein
CL: cardiolipin
CMT: Charcot–Marie‐Tooth
DRP1: dynamin‐related protein1
ER: endoplasmic reticulum
FAD: familial AD
Fis1: fission protein 1
FUNDC1: FUN14 domain‐containing protein 1
FUS: fused in sarcoma
GRAMD1C: GRAM domain containing 1C
GRP75: glucose‐regulated protein 75
GSK3β: glycogen synthase kinase 3β
HD: Huntington's disease
HTT: huntingtin protein
Insig: insulin‐induced gene
InsP3: 1,4,5 inositol trisphosphate
IP3Rs: inositol 1,4,5‐triphosphate receptors
IRE1: immunoglobulin‐regulated enhancer 1
LRRK2: leucine rich repeat kinase 2
MAMs: mitochondria‐associated membranes
MARCH5: membrane‐associated RING‐CH5
MAVS: mitochondrial antiviral‐signaling protein
MCS: membrane contact sites
MERCS: mitochondria‐endoplasmic reticulum contact sites
Mff: mitochondrial fission factor
MFN1: mitofusin1
MFN2: mitofusin2
mHTT: mutant HTT
MiD49: mitochondrial dynamic proteins of 49
MiD51: mitochondrial dynamic proteins of 51
NLRP3: pyrin domain‐containing protein 3
Nrf2: nuclear factor‐erythroid 2‐related factor 2
OMM: outer mitochondrial membrane
OPA1: optic atrophy 1
ORP5: oxysterol‐binding protein‐related proteins 5
ORP8: oxysterol‐binding protein‐related proteins 8
OSBP: oxysterol binding protein
PA: phosphatidic acid
PARP: poly ADP‐ribose polymerase
PC: phosphatidylcholine
PD: Parkinson's disease
PE: phosphatidylethanolamine
PEMT2: phosphatidylethanolamine N‐methyltransferase 2
PERK: immunoglobulin‐regulated enhancer 1
PG: phosphatidylglycerol
PINK1: PTEN‐induced putative kinase 1
polyQ: polyglutamine
PS: phosphatidylserine
PS1: presenilin1
PS2: presenilin2
PSD: phosphatidylserine decarboxylase
PSS: phosphatidylserine synthases
PTPIP51: protein tyrosine phosphatase‐interacting protein‐51
REEP1: receptor expressionenhancing protein 1
RIG‐I: RNA helicase retinoic acid inducible gene I
ROS: reactive oxygen species
RyRs: ryanodine receptors
Sig‐1R: Sigma‐1 receptor
SNPs: single‐nucleotide polymorphisms
SOD1: superoxide dismutase 1
STX17: syntaxin 17
TBI: traumatic brain injury
TBK1: TANK‐binding kinase 1
TDP‐43: TAR DNA binding‐protein 43
TG2: transglutaminase type 2
TIM23: translocator of the inner mitochondrial membrane
TOM70: translocase of outer mitochondria‐70
UPR: unfolded protein response
UPRmt: mitochondrial unfolded protein response
VAPB: vesicle‐associated membrane proteinassociated protein B
VCP: valocin‐containing protein
VDAC: voltage‐dependent anion channels

## 1 Introduction

The World Health Organization has announced that neurodegenerative diseases will pose the most significant health challenges in the twenty‐first century [1]. These diseases have emerged as a widespread and increasingly significant cause of both mortality and morbidity globally, especially among the older population [2]. Unfortunately, for the majority of neurodegenerative diseases, effective treatments remain elusive [3]. Recently, some research has shown that neurons heavily rely on a special area called mitochondria‐associated membranes (MAMs) for signaling molecules and metabolite exchange between organelles [4].

MAM refers to vital interfaces between the mitochondrial outer membrane and endoplasmic reticulum (ER) membrane which play a key role in regulating intracellular signaling and cellular metabolism [5]. As an outstanding platform for crosstalk between the ER and mitochondria, MAM plays a crucial role in diverse signaling pathways that facilitate swift exchange of biological molecules, thereby keeping cellular healthy [6]. Studies have shown that MAMs have significant effects on a variety of cellular processes, including Ca^2+^ signal transduction, lipid metabolism, inflammation, autophagy, apoptosis, and ER stress [7, 8]. Notably, there is a significant preponderance of genes linked to mitochondrial and ER homeostasis in numerous inherited neurodegenerative disorders, emphasizing the crucial role of MAMs in neuronal function [4]. This provides a new idea for the treatment of neurodegenerative diseases. This review will focus on the structural composition and function of MAMs. In addition, it will summarize various neurodegenerative diseases and their relationship with MAMs.

## 2 Structural Composition of MAMs

The initial measurement revealed a distance of approximately 100 nm between the ER and mitochondria [9]. With the development of microscope technology, the first electron microscopy analysis revealed a distance ranging from approximately 10–30 nm [10]. Research indicates that a distance of < 30 nm between the two organelles suggests their connection [11]. MAMs are not formed by membrane fusion [12]. Instead, they use protein tethers to bring the membranes close together, allowing signaling molecules to move quickly and directly between the two compartments [12]. The distance between the ER and mitochondria within MAMs varies based on their specific function [13]. For example, in MAMs involved in Ca^2+^ exchange, the distance ranges from 10 to 25 nm, conducive to accommodating Ca^2+^ channels [14]. When ER tubules participate in mitochondrial fission, they enclose mitochondria at approximately 30 nm, exhibiting a distinct spatial arrangement [15]. In the 1990s, Vance et al. were the first to isolate MAMs from mouse liver by a special method and investigate its biochemical functions [16]. MAMs are composed of the outer membrane of mitochondria and adjacent subdomains of the ER [17]. These structures are made up of phospholipids and display dynamic fluctuations due to their high fluidity [17, 18]. Numerous research studies show that the narrow gap between the ER and mitochondria hosts numerous proteins [19]. The MAM executes its duties through these proteins as its primary means [8]. The initial comprehensive proteome analysis of MAMs was conducted by Zhang et al., revealing approximately 991 proteins within the MAM fraction [20]. Subsequently, Poston et al. expanded the pool of candidates to 1212, encompassing even weakly soluble proteins present at the MAM [21]. In addition to modulating MAM physical interaction, these proteins located on MAMs also play a significant role in regulating the tethering complexes in MAM [22]. There are four major tethering complexes in MAMs of mammalian cells [23].

### 2.1 VAPB‐PTPIP51

**FIGURE 1:**
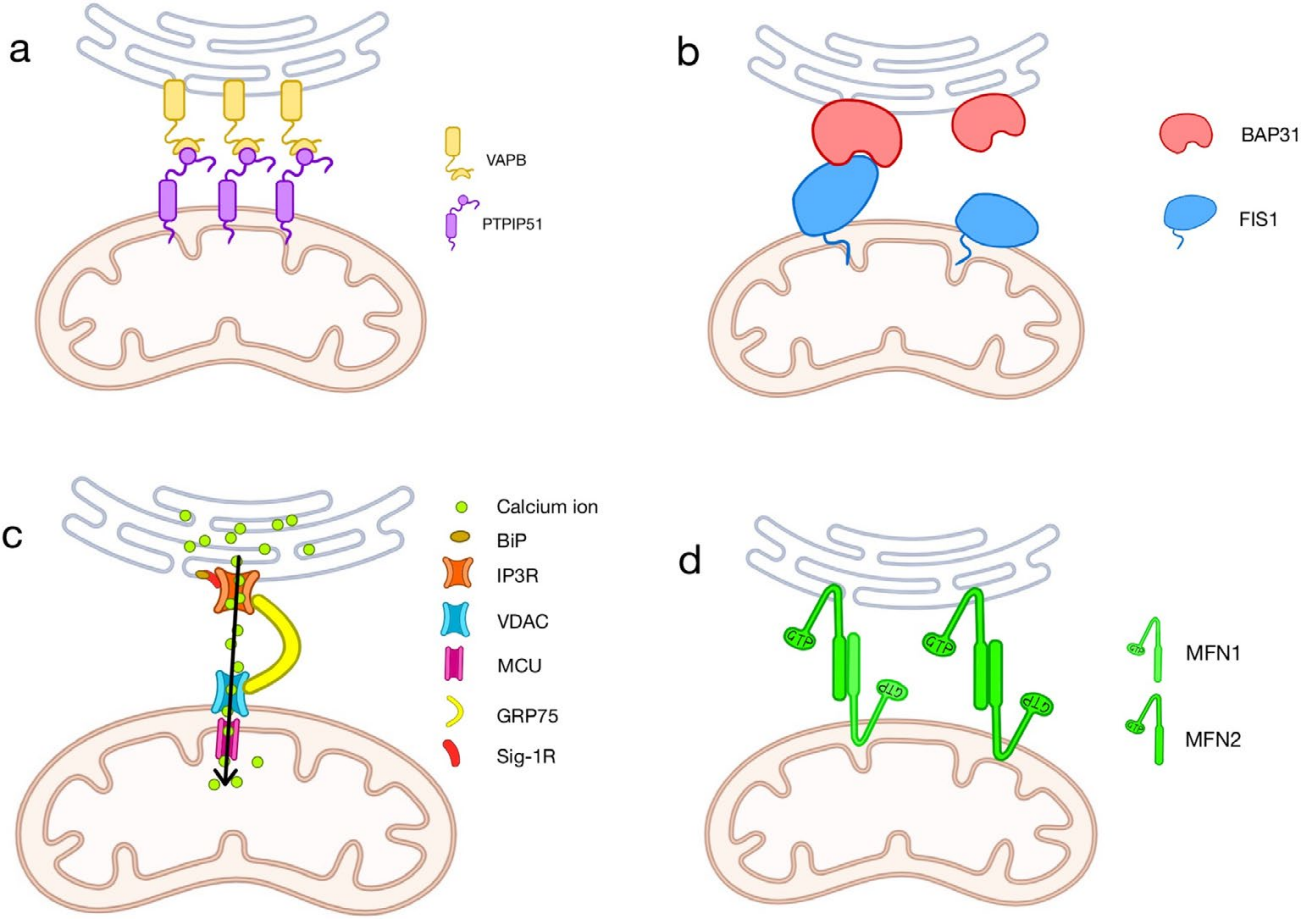
Four major tethering complexes in MAMs of mammalian cells. There are many complex proteins in MAMs. Four of the most important complexes are VAPB‐PTPIP51, BAP31‐Fis1, IP3R‐GRP75‐VDAC, and MFN2‐MFN1. These four tethering complexes have different functions, but they all play a role in neurodegenerative diseases.

Vesicle‐associated membrane protein‐associated protein B (VAPB), a membrane protein associated with vesicles in the ER, interacts with protein tyrosine phosphatase‐interacting protein‐51 (PTPIP51), a protein found on the outer mitochondrial membrane (OMM; Figure 1a) [24]. Together, they form the VAPB‐PTPIP51 tethering complex, which plays a crucial role in regulating the transmission of Ca^2+^ between the ER and mitochondria, mitochondrial adenosine triphosphate (ATP) production, bioenergetics, lipid synthesis, autophagy, and synaptic activity [25, 26, 27].

### 2.2 BAP31‐ Fis1

During apoptosis, B‐cell receptor‐associated protein 31 (BAP31), a protein located in the ER, interacts with the mitochondrial fission protein 1 (Fis1) on the OMM, serving as a tether for MAMs and triggering the apoptotic process (Figure 1b) [28]. The BAP31‐Fis1 complex plays a pivotal role in recruiting and activating procaspase 8, thereby facilitating the transmission of proapoptotic signals from mitochondria to the ER [23].

### 2.3 IP3R‐ GRP75—VDAC

The tripartite complex formed by the ER Ca^2+^ channel inositol 1,4,5‐triphosphate receptors (IP3Rs), the OMM voltage‐dependent anion channels (VDACs), and the cytoplasmic chaperone glucose‐regulated protein 75 (GRP75) regulates the juxtaposition of ER and mitochondria (Figure 1c) [29]. GRP75 oversees the interaction between IP3Rs and VDAC1, ensuring the conformational stability of IP3Rs involved in facilitating Ca^2+^ transport from the ER to mitochondria [30]. The Sigma‐1 receptor (Sig‐1R) interacts with other ER chaperones, such as the binding immunoglobulin protein (BiP), via the IP3R‐VDAC complex [31]. Through this interaction, Sig‐1R helps regulate various functions by supporting the formation of Ca^2+^‐sensitive complexes, which strengthen ionic signaling between the ER and mitochondria [31].

### 2.4 MFN2‐MFN1

Mitofusin2, also known as MFN2, was initially characterized as a dynamin‐like protein that plays a crucial role in the fusion of the OMM [32]. This protein is integral to mitochondrial fusion processes and contributes significantly to the preservation of the mitochondrial network [33]. ER‐resident MFN2 creates either homodimer or heterodimer connections with mitofusin1 (MFN1) or itself on the OMM (Figure 1d) [34]. MFN1 and MFN2 possess overlapping yet distinct functionalities, operating within three distinct molecular complexes to facilitate mitochondrial fusion [35].

## 3 Functions of MAMs

### 3.1 Lipid Metabolism and Transportation

The MAM plays a pivotal role in numerous lipid metabolic pathways [13]. Phosphatidylethanolamine (PE), phosphatidylglycerol (PG), and cardiolipin (CL) are produced within the mitochondria, yet their precursors and a majority of mitochondrial phospholipids undergo synthesis in the ER prior to being transferred to the mitochondria [36]. Phosphatidylserine (PS) is generated through the action of phosphatidylserine synthases (PSS) in the MAM, and it is then transformed into PE by PS decarboxylase (PSD) within the mitochondria [37]. PE is then transformed into phosphatidylcholine (PC) by some enzymes including phosphatidylethanolamine N‐methyltransferase 2 (PEMT2), which was discovered to be exclusively located within the MAMs [38, 39, 40]. In addition, MAMs are rich in other proteins that participate in lipid metabolism, such as fatty acid CoA ligase 4, acyl coenzyme A: cholesterol acyltransferase (ACAT), and diacylglycerol acyltransferase 2 [41, 42].

**FIGURE 2:**
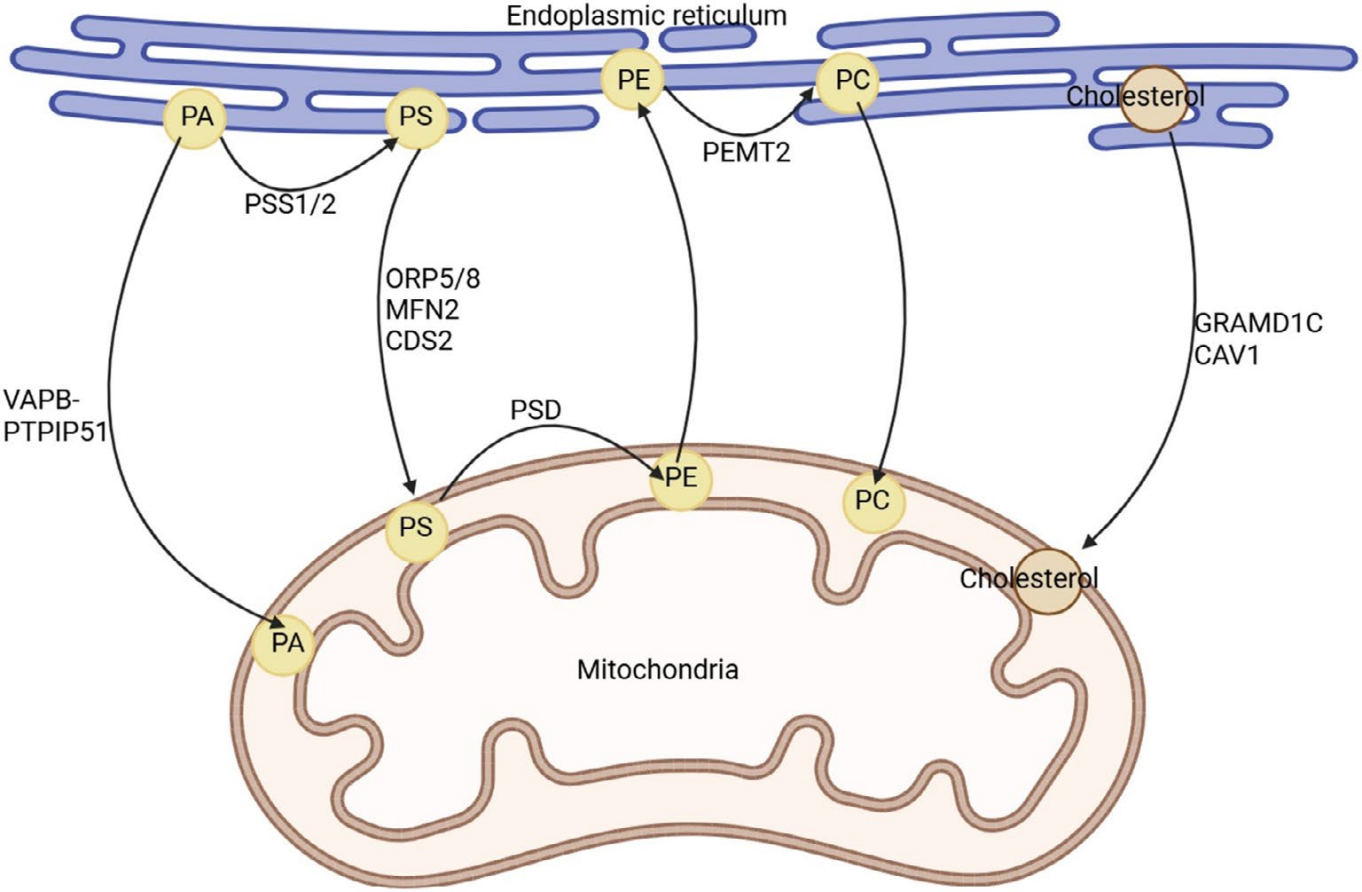
MAM in lipid metabolism and transportation. PA is transformed to PS by PSS in the ER. PS is transferred from the ER to mitochondria with the action of ORP5/8, MFN2, and CDS2. Then, it can be transformed to PE by PSD in mitochondria. PE moves to the ER and is transferred to PC by PEMT2. Finally, PC can be transformed to mitochondria again. VAPB‐PTPIP51 also plays a role in the movement of PA. GRAMD1C and CAV1 are proved to take part in the movement of cholesterol from the ER to mitochondria.

Vance et al. demonstrated that apart from vesicle transportation, lipid transport between organelles may also occur through protein‐facilitated reactions or tight junctions between membranes [43]. MAMs create a hydrophilic interface between the ER and mitochondria, fostering the bidirectional and nonvesicular exchange of lipids [42]. Studies have pinpointed relative proteins situated in the MAM, namely oxysterol‐binding protein‐related proteins 5 and 8 (ORP5/8), MFN2, and CDP‐diacylglycerol synthase‐2 (CDS2), which facilitate the nonvascular transfer of PS from the ER to mitochondria [44, 45, 46]. CL undergoes a series of modifications derived from phosphatidic acid (PA), and research indicates that the VAPB‐PTPIP51 complex plays a crucial role in regulating the transfer of PA within the MAMs [47]. GRAM domain containing 1C (GRAMD1C) and caveolin‐1 (CAV1), both located in the MAM, have also been shown to be involved in the transport of lipids between membranes [48, 49]. All these processes are summarized in Figure 2.

### 3.2 Ca^2+^ Transportation

MAMs act as central hubs for Ca^2+^ signaling [50]. They enable the transfer of Ca^2+^ from the ER, which is a key storage site for this ion, to mitochondria through the physical contact points mediated by MAMs [50]. MAM harbors two distinct calcium‐releasing channels: ryanodine receptors (RyRs) and IP3Rs [51]. Although RyRs have been shown to play a role in neurodegenerative diseases, studies on its association with MAMs have been lacking [52]. The IP3R, which is abundantly present at ER‐mitochondrial contact sites, is considered the key Ca^2+^ channel facilitating the release of Ca^2+^ from the ER to mitochondria [37]. Upon activation, IP3R on the ER membrane releases Ca^2+^ from the lumen [8]. With GRP75's aid, Ca^2+^ is then sequestered by VDAC1 on the OMM, traverses the MCU of the IMM, and forms a Ca^2+^ signaling cascade within MAMs [8]. Besides the previously mentioned proteins, MAMs contain numerous other proteins vital to Ca^2+^ transport. Glycogen synthase kinase 3β (GSK3β) modulates mitochondrial Ca^2+^ homeostasis in cardiomyocytes through its interaction with IP3Rs [53]. Cyclophilin D plays a crucial role in maintaining cardiac function by regulating calcium dynamics in these cells [54]. Sig‐1R, a non‐G‐protein coupled chaperone of the ER, preserves the stability of IP3R, also ensuring effective Ca^2+^ signaling between the ER and mitochondria [55]. Moreover, calnexin participates in protein folding while also engaging with ER Ca^2+^ pumps [56]. Besides, studies have shown that the distance between the ER and mitochondria can influence Ca^2+^ transport and organelle function [57]. A 15 nm increase in this distance enhances Ca^2+^ transport efficiency, whereas a reduction to 5 nm results in decreased efficiency [58].

### 3.3 Mitochondrial Dynamics

Mitochondrial dynamics, encompassing both fission and fusion, are essential for upholding cellular homeostasis [18]. MAM regulates mitochondrial morphology, including shape, size, and length, by balancing mitochondrial fusion and fission processes [59]. Mitochondrial fission is regulated by motility‐related GTPase proteins, including dynamin‐related protein1 (DRP1) and its receptors Fis1, mitochondrial fission factor (Mff), as well as mitochondrial dynamic proteins of 49 and 51 (MiD49 and MiD51), which together control the process [60]. Drp1 resides primarily in the cytoplasm and is summoned to ER‐mitochondrial contact sites via receptors like Mff, Fis1, and MiD49/51 [61, 62]. There, it assembles into a helical oligomer, triggering membrane constriction and scission [61, 62]. Mitochondrial fusion necessitates synchronized outer and inner membrane fusion processes, primarily controlled by three GTPases: MFN1, MFN2, and optic atrophy 1 (OPA1) [63]. MFN2 can form dimers with either MFN1 or MFN2 present on the mitochondria, thereby facilitating the fusion of the OMM of mitochondria [35]. The pro‐apoptotic Bcl2 protein Bax also interacts with MFN2 to enhance mitochondrial fusion in healthy cells [64]. In this process, the soluble, monomeric form of Bax regulates the assembly of MFN2 complexes at the sites of mitochondrial fusion [64]. Some other MAM‐associated molecules also play a significant role in regulating mitochondrial dynamics, such as syntaxin 17 (STX17), the ER‐anchored isoform of the formin protein inverted formin 2, phosphofurin acidic cluster sorting protein 2, FUN14 domain‐containing protein 1 (FUNDC1), and so on [65, 66, 67].

### 3.4 Autophagy and Apoptosis

A significant number of autophagic proteins reside in MAMs, and it is possible that autophagosomal membranes are derived from these structures [18]. After autophagy induction, the preautophagosome marker autophagy‐related gene 14‐like (ATG14L) and omegaome marker double FYVE domain‐containing protein 1 localize to the MAMs again to stimulate the formation of autophagosome [68]. Additionally, ATG5, another marker of autophagosome formation, also relocates to the MAMs following starvation [69]. It has been reported that the recruitment of ATG5 and ATG14 is regulated by soluble N‐ethylmaleimide‐sensitive factor attachment protein receptor protein STX17 [69]. The tethering complex VAPB‐PTPIP51 also plays a role in regulating autophagy. Increasing the expression of VAPB or PTPIP51 can decrease autophagosome formation by strengthening the connections between the ER and mitochondria [24]. Conversely, decreasing these contacts by suppressing VAPB or PTPIP51 expression can promote autophagosome formation [24]. Moreover, during hypoxic conditions, key enzymes involved in mitophagy, including PTEN‐induced putative kinase 1 (PINK1), recombinant beclin 1 (BECN1), PARK2, and FUNDC1, accumulate at the MAM to initiate autophagosome formation, thereby facilitating the removal of damaged mitochondria [70, 71].

Tethering complex BAP31‐ Fis1 and IP3R‐ GRP75—VDAC in MAMs both contribute to apoptosis [72]. The protein Fis1 on the OMM plays a crucial role in recruiting DRP1 to sites of mitochondrial division [73]. Meanwhile, the ER chaperone BAP31 modulates the degradation of misfolded proteins and the apoptotic pathway [74]. The apoptotic signal originating from mitochondria is transmitted to the ER via Fis1's physical interaction with BAP31, triggering caspase‐dependent cleavage of BAP31 [75]. This cascade leads to the activation of procaspase‐8, which moves to the MAM by the Fis1‐BAP31 tethering complex [75]. Effectively suppressing IP3R expression attenuated apoptosis triggered by both extrinsic and intrinsic apoptotic pathways [76]. Analogously, downregulation of VDAC1 expression specifically rescued the apoptotic process initiated by low‐amplitude apoptotic Ca^2+^ signal transduction [77].

### 3.5 ER Stress

An imbalance between the protein folding demands and the ER's folding capacity, resulting from physiological needs or disease conditions, can trigger ER stress [78]. This results in the accumulation of unfolded or misfolded proteins within the ER lumen, triggering the activation of the unfolded protein response (UPR) [11]. UPR is a conserved adaptive pathway that enables the recovery of normal functions in organelles, even when misfolded proteins accumulate in them [79]. The pathway is primarily mediated by the ER‐resident sensor proteins immunoglobulin‐regulated enhancer 1 (IRE1), protein kinase R‐like endoplasmic reticulum kinase (PERK), and activating transcription factor 6 (ATF6), which are normally maintained in an inactive state by GRP78 [80]. Studies show that the lack of PERK reduces endogenous apoptosis caused by ER stress [81]. This is because PERK deficiency hinders MAM formation and interferes with the transmission of ROS signals to nearby mitochondria [81]. IRE1's presence in MAMs determines IP3R's effectiveness in facilitating Ca^2+^ transfer to mitochondria [82]. These mechanisms bridge the gap between ER stress and mitochondrial function, ultimately influencing cellular fate.

### 3.6 Mitochondrial Stress

To uphold cellular stability and prevent stress‐related damage, mitochondria trigger a series of reactions termed mitochondrial stress responses. These include the dynamic processes of fission and fusion, mitophagy, mitochondrial unfolded protein response (UPRmt), and antioxidant defenses [83]. We have already mentioned fission, fusion, and mitophagy before. Now, we will focus on UPRmt and antioxidant defenses. The synchronization of UPR signaling with energetic requirements occurs at MAMs, which are specialized regions facilitating communication between organelles [84]. Studies have demonstrated that MAMs transmit stress signals from the ER to mitochondrial targets through UPR pathways, particularly when ER protease homeostasis is disrupted [85]. During the UPR's adaptive phase, ER‐mitochondrial interactions increase ATP production by facilitating Ca^2+^ transfer through MAMs [84]. Additionally, numerous other UPR pathway components and calcium‐regulating factors are situated at the MAMs. For antioxidant defenses, studies have shown that MAMs exerts an indirect antioxidant effect by regulating Ca^2+^ flow and lipid metabolism to maintain mitochondrial function [86]. Take for example, Sig‐1R located in MAMs exhibits antioxidant properties by regulating Ca^2+^ and lipid transport, effectively decreasing lipid peroxidation [87]. Proteins in MAMs, such as STARD7, are also involved in the synthesis and transport of coenzyme Q (CoQ), which is an important component of the mitochondrial antioxidant defense system [88]. Moreover, in Alzheimer's disease, the MAM reduces oxidative stress and delays disease progression by regulating the communication between mitochondria and ER [19].

### 3.7 Inflammation

Tschopp et al. observed that ROS promotes the activation of the NLRP3 inflammasome, a member of the NOD‐like receptor family [89]. This finding connects the ER‐mitochondria interface to the regulation of inflammatory responses [89]. The MAM was discovered to play a dual role, not only participating in the activation of inflammation but also serving as a hub for NLRP3 inflammasome assembly [90]. In its resting state, NLRP3 resides in the cytoplasm and within the ER [90]. After stimulation, the NLRP3 inflammasome is recruited to MAM sites together with its adaptor apoptosis‐associated speck‐like protein containing a CARD (ASC), indicating that NLRP3 accumulates strategically at mitochondria to detect mitochondrial damage [89]. Moreover, the distribution of the VDAC protein across MAM holds significant implications for the inflammatory response [11]. VDAC1 plays a pivotal role not just in facilitating the mitochondrial uptake of Ca^2+^ but also in augmenting the generation of ROS [89]. Notably, the downregulation of VDAC1 markedly suppresses the formation of the IL‐1β inflammasome, further emphasizing its crucial function in the inflammatory cascade [89].

### 3.8 Regulation of Antiviral Signaling

Upon infection with an RNA virus, the cytosolic RNA helicase retinoic acid inducible gene I (RIG‐I) receptor, responsible for recognizing pathogens, is recruited to the junction between the ER and mitochondria [91]. There, it recruits its adaptor protein mitochondrial antiviral‐signaling protein (MAVS), initiating an intracellular immune hub that triggers the body's natural defenses against the virus [91]. Moreover, Michael Gale Jr.'s group conducted proteomic analysis, revealing the localization of novel proteins to MAMs during RNA virus replication, including members of the RAS oncogene family 1B, vitronectin, and Lon peptidase 1 [92].

## 4 MAMs in Neurodegenerative Diseases

### 4.1 Alzheimers' Disease

Alzheimer's disease (AD) remains the most prevalent cause of dementia, making up a significant portion (80%) of dementia diagnoses [93]. It stands as the primary cause of dementia, swiftly emerging as one of the most costly, fatal, and burdensome illnesses of this century [94]. The disease typically begins with mild memory problems [95]. Over time, it progresses to more noticeable cognitive decline, difficulty performing complex daily tasks, and impairments in other cognitive functions [95]. Alzheimer's currently affects more than 55 million people worldwide [96].

The aberrant cleavage of the amyloid precursor protein (APP) serves as a key indicator of AD pathogenesis [4]. Key contributors to familial AD (FAD) are mutations in presenilin1 (PS1) and presenilin2 (PS2), integral components of the γ‐secretase complex that is instrumental in APP processing [97]. Research has demonstrated that MAMs serve as a hub for key proteins implicated in AD pathogenesis [96]. One reason is that both PS1 and PS2 are highly concentrated in MAMs [96]. Additionally, ACAT, an enzyme thought to play a key role in Aβ production, is also mainly located in these membrane compartments [96]. Eric Schon's team initially established the concentration of presenilins in the MAM, proposing it as the primary subcellular locale for PS1/PS2 and γ‐secretase activity [72]. Subsequently, they discovered a marked elevation in MAM functionality and ER‐mitochondrial communication in cells lacking presenilin as well as in fibroblasts derived from patients with both familial and sporadic forms of AD [72]. When PS1 and PS2 mutate, these proteins trigger the opening of IP3R channels, leading to an influx of intracellular Ca^2+^ and ultimately stimulating the production of Aβ [98]. Moreover, Area‐Gomez et al. demonstrated that APP is also enhanced and active in a MAM‐enriched subcellular fraction [99].

Sig‐1R also plays a significant role in Alzheimer's disease. Although Sig‐1R levels remain relatively stable during normal aging, it has been observed that there is a decrease in Sig‐1R levels in the brains and postmortem tissues of AD patients [100]. The interaction between Sig‐1R and insulin‐induced gene (Insig) may play a significant role in this disease, given that Insig has been implicated in disease progression through its influence on cholesterol synthesis [101]. Sig‐1R plays a crucial role in preserving the structural integrity of the MAM, presumably by anchoring it to IP3R and VDAC [102]. An increase in the expression of IP3R and VDAC has been observed in brain samples from AD patients [102]. VDAC is a key protein that interacts with more than 150 other proteins, such as phosphorylated tau, Aβ, and γ‐secretase [103]. These interactions may enhance their harmful effects, potentially triggering cell death and mitochondrial dysfunction during the development and progression of AD [103]. The functional impairment of Sig‐1R also plays a crucial role in neuronal loss triggered by β‐amyloid (Aβ) [104]. As the central component of AD pathology, Aβ is composed of self‐aggregated peptides containing 40–42 amino acids derived from the proteolysis of APP, a membrane protein ubiquitously expressed in the central nervous system [105]. In the amyloidogenic process, APP is initially cleaved by β‐secretase, releasing a soluble APPβ fragment and C99 [79]. Subsequently, γ‐secretase cleaves C99, generating the APP intracellular domain and Aβ [79]. In healthy neurons, C99 is cleaved to generate Aβ40 (40 amino acids long) [106]. However, in neurons affected by AD, C99 is cleaved to produce Aβ42 (42 amino acids long), leading to an increased ratio of Aβ42 to Aβ40 [106]. This accumulation of Aβ42 in plaques can cause cellular toxicity and result in abnormal hyperphosphorylation of tau [106]. APP and β‐secretases in this process are present in MAMs, where they engage in APP processing activities [107]. Enhanced expression of mutant APP or exposure to nanomolar levels of Aβ elevates the number of ER‐mitochondria contact points and mitochondrial Ca^2+^ concentrations [102, 107]. The interaction of Ca^2+^ with β‐site amyloid precursor protein cleaving enzyme 1 (BACE1) enhances its proteolytic activity, leading to increased Aβ peptide production, exacerbation of Aβ formation, and promotion of tau hyperphosphorylation [98]. Certain genetic combinations of Sig‐1R and apolipoprotein E (Apo‐E) genotypes can also interact synergistically to elevate the risk of AD [108]. Tambini et al. proposed that ApoE4 secreted by astrocytes notably enhances ER‐mitochondrial communication and MAM function, as indicated by the synthesis of phospholipids and cholesteryl esters [109].

**FIGURE 3:**
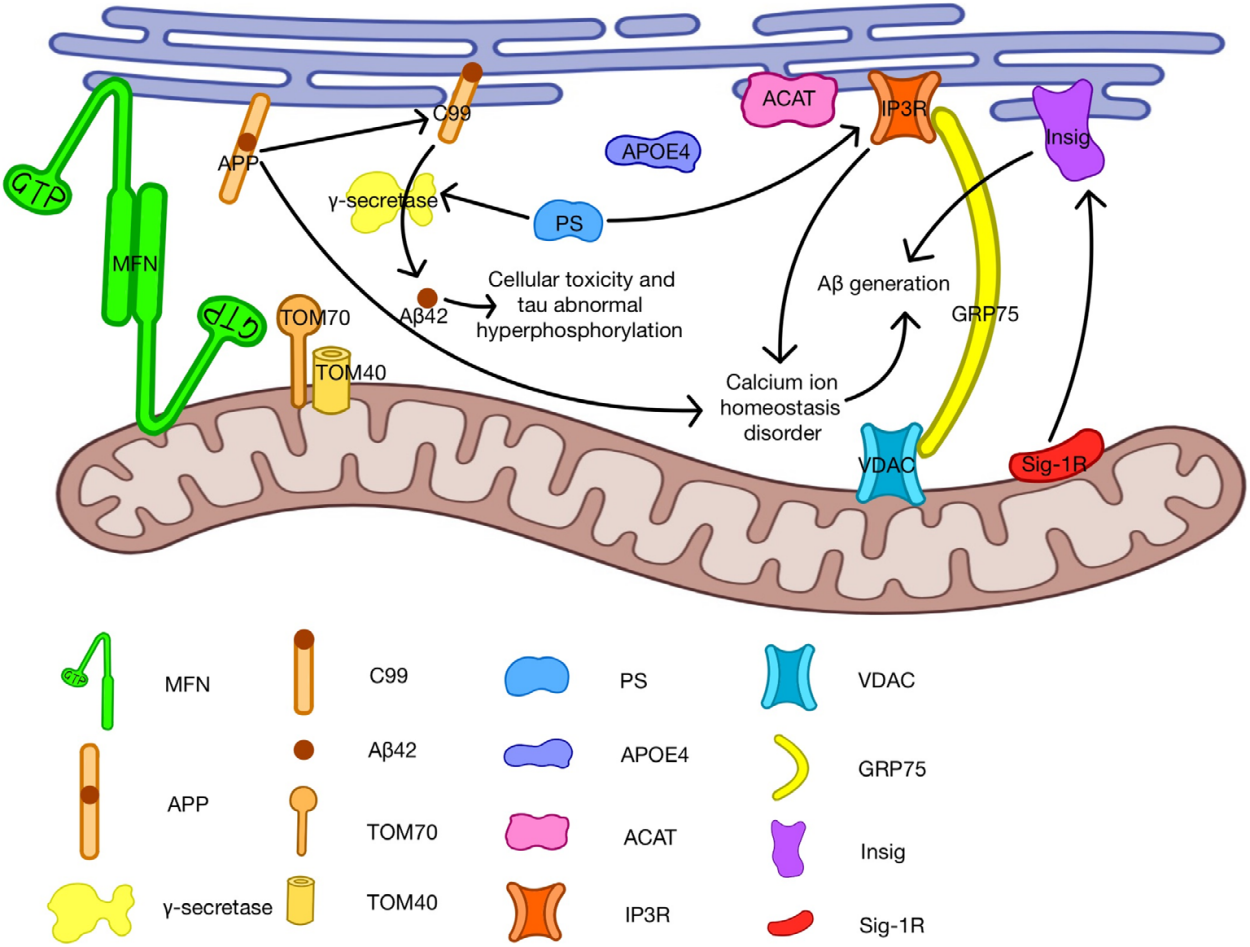
The influence of MAMs on Alzheimer's disease. The effect of MAM on Alzheimers' disease involves multiple pathways. (1). Mutation of APP will lead to an increase of ER‐mitochondria contact points, which can cause Ca^2+^ homeostasis disorder and increase Aβ generation. (2). Mutation of PS1/PS2 will open IP3R, which also causes Ca^2+^ homeostasis disorder and increases Aβ generation. (3). As components of the γ‐secretase, PS can influence the production of Aβ42. The abnormal ratio of Aβ42 to Aβ40 will affect cellular toxicity and hyperphosphorylation of tau. (4). Sig‐1R can influence Insig, which will cause cholesterol metabolism imbalance and lead to Aβ generation. (5). APOE4, ACAT, TOM70, and some other proteins also play a significant role in Alzheimer's disease.

Moreover, some other MAM‐related proteins have also been implicated in Alzheimer's disease. For example, MFN1, MFN2, and translocase of outer mitochondria‐70 (TOM70) are essential proteins in mitochondria‐ER contact sites (MERCS), with their levels being decreased in FAD cases [110]. Elevated levels of reactive oxygen species (ROS) could also potentially contribute to the onset of AD [110]. All these mechanisms and proteins involved are summarized in Figure 3.

A major reason for the lack of effective Alzheimer's disease treatments is our limited understanding of the specific molecular and cellular mechanisms that cause the disease [79]. MAMs occupy a crucial role in AD pathogenesis, making them a promising therapeutic target. Many studies have shown that GSK3β, a key brain homeostasis regulator implicated in numerous disorders including AD, acts as a negative modulator of ER‐mitochondria membrane contact sites formation, presenting itself as a potential therapeutic target [111, 112, 113]. Besides, drugs that activate Sig‐1R are regarded as neuroprotective and anti‐amnestic agents in neurodegenerative diseases, including AD [104]. For example, a recent study showed that N, N‐dimethyltryptamine, a natural hallucinogenic drug, may improve AD by restoring ER‐mitochondrial crosstalk mediated by neurons' Sig‐1R [114]. Moreover, emerging research proposes a unique mechanism linking transglutaminase type 2 (TG2) brain activation with the regulation of Aβ‐induced Ca^2+^ signaling and MAM function in AD [115]. Hence, future studies might explore the modulation of TG2 activity as a promising therapeutic approach. In addition, Rer1 has been identified as a candidate protein for treating glucocorticoid‐triggered Aβ production in mitochondria [116]. At present, there are many studies on Aβ, but there are few studies on tau. In the future, we can conduct more in‐depth research on tau to find newer therapeutic targets for Alzheimer's disease.

### 4.2 Parkinson's Disease

James Parkinson, a British physician, initially described Parkinson's disease (PD) in 1817 [117]. With a global prevalence exceeding 6 million individuals, this disease ranks as the second most common neurodegenerative disease [117]. Its occurrence has increased dramatically, with a 2.5‐fold rise in prevalence over the past generation [118]. As a result, it has become a leading cause of neurological disabilities worldwide [118]. PD is a disease related to age. Global statistics indicate that 1%–2% of individuals aged 65 and older, and 4%–5% of those over 85, are afflicted with PD [119]. Furthermore, the occurrence of this disease rises by 5–10 times among those aged 60–90 [119]. While most PD cases occur sporadically, there have been instances of familial cases associated with genetic mutations in specific genes, known as PARK genes [120]. These genes encode proteins such as α‐synuclein (PARK1/4 or SNCA), parkin (PARK2), PINK1 (PARK6), DJ‐1 (PARK7), leucine‐rich‐repeat kinase 2 (LRRK2, PARK8), and others [120].

One of the crucial pathophysiology features of PD is the presence of Lewy bodies, which are composed of aggregated α‐synuclein [121]. In 2013, Poston et al. analyzed proteins from mouse brains and detected α‐synuclein in MERCS [21]. Guardia‐Laguarta et al. later confirmed this, suggesting that α‐synuclein is specifically found in MERCS, not mitochondria as previously thought [122]. Since MAM regulates many cellular processes, studying α‐synuclein's presence and role in MAM will improve our understanding of its functions and its damaging effects in Parkinson's disease. Wild‐type α‐synuclein, with its strong affinity for lipid rafts, localizes to MAMs, facilitating physical interactions with mitochondria [123]. Contrary to wild‐type α‐synuclein, mutant forms of α‐synuclein that cause PD lead to a downregulation of ER‐mitochondrial apposition, resulting in reduced MAM function and increased mitochondrial fragmentation [122]. A30P and A53T are the mutant forms of this protein [37]. The A30P mutation modifies the interaction between SNCA and rafts, while the A53T mutation leads to a reduced amount of SNCA, resulting in a lower association accordingly [37]. Overexpression and mutation of α‐synuclein will further cause disruptions in Ca^2+^ homeostasis between organelles, leading to elevated mitochondrial ATP production [124]. Moreover, synaptic dysfunction is also a prominent aspect of PD [125]. A study revealed that α‐synuclein can interact with VAPB, disrupting the VAPB‐PTPIP51 interaction and ultimately leading to reduced mitochondrial ATP production [125]. Research also shows that α‐synuclein can interfere with the expression of the IP3R‐GRP75‐VDAC complex, blocking Ca^2+^ transfer between the ER and mitochondria [126]. Remarkably, neurons from familial PD patients with pathogenic triplication of α‐synuclein show this disruption [125]. Furthermore, the presence of SNCA and its function in MAMs may affect cholesterol regulation in PD, due to MAM dysfunction resulting from SNCA mutations [122].

Mutations that disable PINK1 and Parkin (an E3 ubiquitin ligase), both proteins essential for mitophagy, underlie familial PD [96]. Both of them localize to the MAM compartment [96]. Research has found increased contacts between mitochondria and the ER in flies and human fibroblasts from PD patients with PINK1 or Parkin mutations [127]. Previous studies have shown that overexpressing Parkin enhances the physical and functional coupling between the ER and mitochondria, boosting ATP production and Ca^2+^ exchange [128]. Conversely, knocking down Parkin has the opposite effect, disrupting mitochondrial morphology [128]. This may be caused by MFN2 in MAMs and agonist generated by 1,4,5 inositol trisphosphate (InsP3) [128, 129]. During mitochondrial and ER stress, Parkin levels increase in an ATF4‐dependent way [130]. This helps regulate the interaction between the ER and mitochondria, promoting cell survival under stress [130]. Moreover, Parkin plays an important role in the ubiquitin–proteasome system [131]. An elevated level of Parkin within the MAM fraction results in ubiquitination of numerous MAM proteins, including VDAC and MFN2 [132]. Therefore, it is noteworthy that in cells lacking Parkin and human fibroblasts with Parkin mutations, the connection between the ER and mitochondria is reduced [98]. PINK1, a protein linked to familial PD and mitochondria quality control, as well as the autophagy‐promoting protein BECN1/Beclin1, were both detected at the MAM [71]. The interaction between PINK1 and Beclin1 strengthened the contact between the ER and mitochondria, facilitating the formation of autophagosomes during mitophagy induction [71]. During mitophagy, PINK1 and Parkin jointly catalyze a swift surge in MFN2 ubiquitination, leading to the detachment of mitochondria from the ER by dismantling MFN2 tethers [98]. As a result, MAMs disintegrate, and the decrease in ER‐mitochondria appositions accelerates the rate of mitochondrial degradation [98]. In addition, the silencing of the PINK1 gene in M17 dopaminergic cells led to a decrease in the number of ER‐to‐mitochondria contacts, resulting in an impaired Ca^2+^ homeostasis [124]. Overexpression of Parkin or PINK1 mutated in familial PD has been demonstrated to trigger the PERK pathway of the UPR of ER and enhance the connectivity between the ER and mitochondria in a mitofusin‐dependent fashion [79, 127]. Furthermore, PINK1/Parkin‐mediated mitophagy is one of the underlying mechanisms leading to dopaminergic neuron death [71]. A study on neurons exposed to glutamate‐induced excitotoxicity revealed that the expression of Parkin increases in the MAM fraction following mitophagy [133]. PINK1 facilitates the proteasomal degradation of Miro1/2 and MFN1/2 in a Parkin‐dependent manner [4]. Miro, a target of PINK1/Parkin, plays a pivotal role in mitochondrial transfer along microtubules [131]. Its dysfunction can contribute to the malfunctioning of mitochondria or ER in Parkinson's disease [131]. Interestingly, the lack of Miro has been shown to counteract the effects of PINK1 mutations linked to Parkinson's disease in Drosophila and helps remove damaged mitochondria in HeLa cells [134]. This highlights its important role in the disease process.

DJ‐1, encoded by the PARK7 gene, is a multifunctional protein encompassing 189 amino acids [30, 124]. Multiple gene mutations in DJ‐1, including deletions and point mutations, have been firmly linked to early autosomal recessive Parkinson's disease [135]. The DJ‐1 protein can also be found at the MAM, affecting its interactions and the flow of Ca^2+^ between the ER and mitochondria [124]. Liu et al. demonstrated that DJ‐1 can interact with MAM and is a key part of the IP3R‐Grp75‐VDAC complexes [136]. Lower DJ‐1 levels in the substantia nigra of PD patients might damage the IP3R‐GRP75‐VDAC tethering protein complex, disrupting Ca^2+^ crosstalk and ATP production, ultimately resulting in mitochondrial structural abnormalities and dysfunction [105]. In addition, the interaction between DJ‐1 and the anti‐oncogene p53 is thought to play a role in PD [137]. Excessive p53 has the opposite effect on ER‐mitochondria Ca^2+^ transfer and ER‐mitochondrial interaction compared to DJ‐1, reducing Ca^2+^ transients and this interaction [138]. However, when DJ‐1 and p53 are expressed together, the impact of p53 overexpression is counteracted [138]. This restores histamine‐induced Ca^2+^ transients to DJ‐1 levels and brings ER‐mitochondrial interactions back to normal, matching control cell levels [138]. Moreover, S‐palmitoylation (a reversible protein post‐translational modification) of DJ‐1 prompts its localization to lipid rafts in astrocytes, potentially contributing to PD pathogenesis [137]. It has also been demonstrated that suppressing DJ‐1 results in elevated levels of aggregated α‐synuclein in PD cells and animal models [105].

**FIGURE 4:**
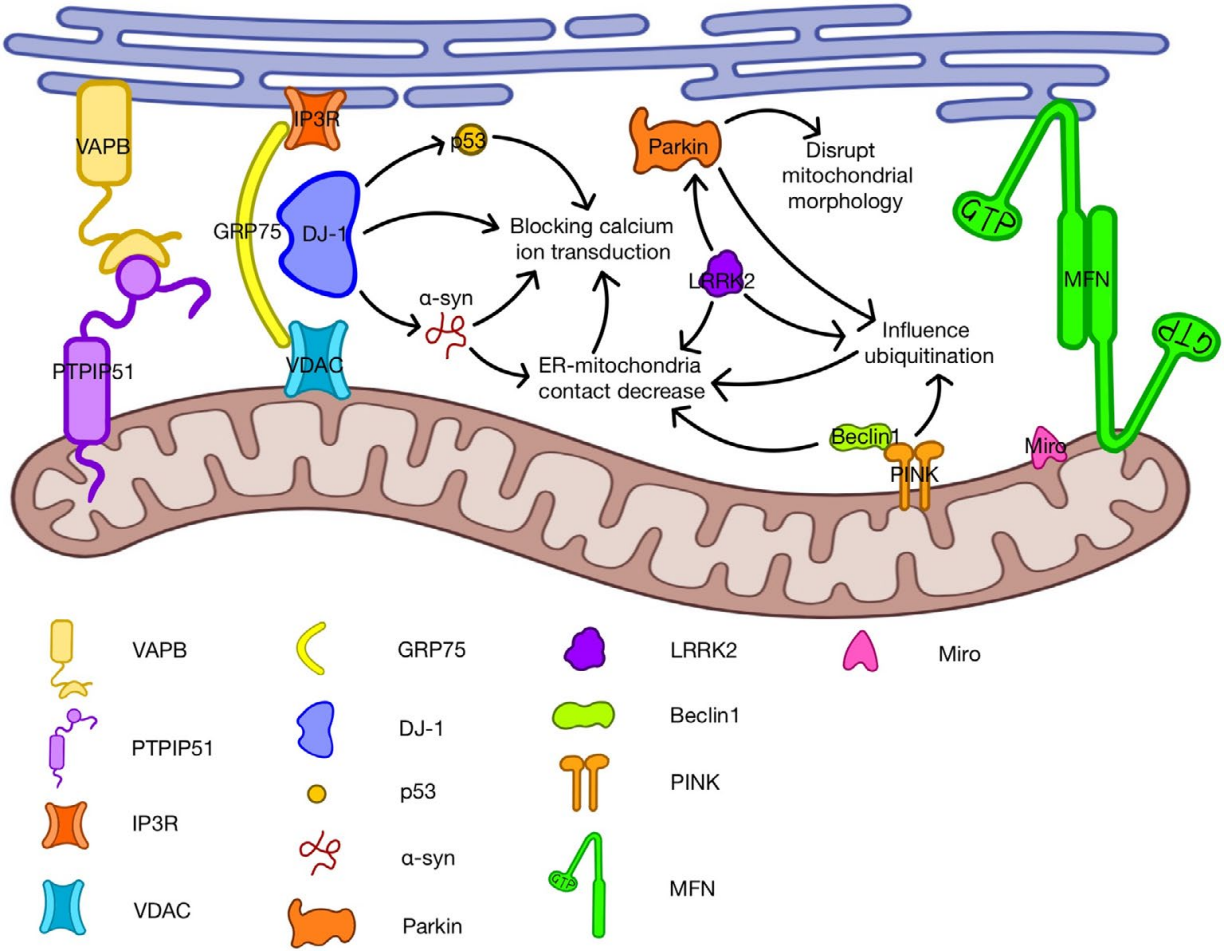
The influence of MAM on Parkinson's Disease. There are five main types of mutations that affect Parkinson's disease in MAMs. They are α‐synuclein, Parkin, PINK1, DJ‐1, and LRRK2. For α‐synuclein, (1) it will bind to or interfere with VAPB, which disrupts contact between the ER and mitochondria. (2) It can cause ER‐mitochondria contact decrease directly. (3) It will block Ca^2+^ transduction through its influence on IP3R‐GRP75‐VDAC. For Parkin: (1) Knocking down Parkin may disrupt mitochondrial morphology. (2) It will influence ubiquitination of protein, including VDAC and MFN2. Then, it will affect ER‐mitochondria contact and blocking Ca^2+^ transduction. (3) It will work together with PINK to increase degradation of Miro1/2 and MFN1/2. For PINK1, (1) it will influence ubiquitination of protein like Parkin. (2) It will interact with Beclin1 to decrease ER‐mitochondrial contact. Then lead to blockage of Ca^2+^ transduction. For DJ‐1, (1) it can impair IP3R‐GRP75‐VDAC which may affect transduction of Ca^2+^. (2) The number of DJ‐1 has effects on the number of P53. An excess of P53 will cause a decrease of Ca^2+^ in MAMs. (3) Disruption of DJ‐1 will elevate levels of aggregated α‐synuclein. For LRRK2, (1) loss of LRRK2 will lead to ER‐mitochondria contact decrease directly. (2) It can affect IP3R‐GRP75‐VDAC and influence Ca^2+^ through phosphorylation and activation of Parkin. (3) It plays a role in ubiquitination of protein through binding to E3 ubiquitin ligases like Parkin.

Mutations in the leucine‐rich repeat kinase 2 (LRRK2) gene are associated with late‐onset autosomal PD [139]. Six significant mutations in the LRRK2 gene have been identified as pathogenic, accounting for 5%–6% of familial PD cases and 1%–2% of sporadic PD cases [139]. In 2020, Toyofuku et al. found that LRRK2 plays a crucial role in the formation and function of MERCS [140]. Specifically, the deletion of LRRK2 led to a decrease in contact sites and a disruption in Ca^2+^ flux to mitochondria [140]. LRRK2 has been shown to control PERK activity, which affects the structure of MERCS [79]. It also regulates the IP3R‐VDAC1‐dependent Ca^2+^ transfer between the ER and mitochondria by phosphorylating and activating Parkin [79]. Studies have shown that the mutated LRRK2 gene binds to E3 ubiquitin ligases like Parkin and membrane‐associated RING‐CH5 (MARCH5), inhibiting their activation in MAM [124]. This prevents the ubiquitination and degradation of MAM components, leading to a reduction in IP3R‐GRP75‐VDAC‐mediated Ca^2+^ export and mitochondrial ATP production [124]. Additionally, overexpression of mutant LRRK2 genes, specifically G2019S and R1441C, leads to enhanced interaction with Drp1, resulting in hyperphosphorylation of fission proteins [141]. This, in turn, increases ROS production and mitochondrial fragmentation [141]. LRRK2 also modulates the activity of proteins involved in mitochondrial fusion, such as MFN1, MFN2, and OPA1 [124]. All these mechanisms and proteins involved are summarized in Figure 4.

There are some other substances related to MAM that play a role in PD. The dopamine receptors and Sig‐1R interactions are also a potential point of interference of MAMs in PD [98]. There is compelling evidence that suggests ER stress could be a supplementary mechanism underlying the pathogenesis of PD [142]. Bioinformatics analysis has revealed the presence of two single‐nucleotide polymorphisms (SNPs) within the genes encoding CAST, an endogenous inhibitor of the calpastatin gene [143]. These SNPs are associated with a predisposition to idiopathic PD, indicating a potential genetic link between these variations and the disease's development [143]. The initial report linking MAMs and neuroinflammation was published by Zhou et al., highlighting the crucial role of mitochondria in NLRP3 inflammasome activation [89]. The presence of angiotensin receptors and the RAS components in the MAM fractions is also supposed to be a potential target for various neurodegenerative diseases, including Parkinson's disease [124]. Moreover, genes involved in lipid and lipoprotein signaling play a role in PD pathogenesis, such as oxidative stress, lysosomal dysfunction, ER stress response, and immune response [137].

Developing treatments to slow or stop Parkinson's disease progression remains a critical focus for both patients and researchers alike, yet no definitive disease‐modifying therapies have been identified to date [144]. Some research suggests that targeting Sig‐1R might be one of the most promising strategies for treating Parkinson's disease [145]. Recent studies reveal that phillyrin boosts MAM formation by increasing receptor expression‐enhancing protein 1 (REEP1) levels [146]. This reduces cardiolipin externalization through the REEP1‐NDPK‐D interaction and strengthens autophagosome production [146]. This mechanism highlights phillyrin's potential as a treatment for Parkinson's disease. Moreover, Jihoon Lee et al. developed a platform called autophagy‐targeting chimera (ATUOTAC), which can facilitate the creation of degraders targeting α‐syn aggregates, presenting a novel therapeutic approach to modify the course of PD [147]. New study also shows that S‐palmitoylation could be a potential therapeutic target in the future [137].

### 4.3 Amyotrophic Lateral Sclerosis

Amyotrophic lateral sclerosis (ALS) is a progressively fatal neuromuscular disease, distinguished by the degradation of both upper and lower motor neurons, ultimately leading to the malfunction of the body's somatic muscles [148]. ALS is a relatively uncommon disorder, with a standardized global incidence rate of only 1.68 cases per 100,000 person‐years of follow‐up, but this figure may vary depending on the region [149]. The majority of ALS patients succumb to respiratory failure within 3 years of diagnosis [150]. ALS manifests both sporadically and as a familial condition, accounting for 10%–15% of cases being familial and 85%–90% being sporadic [151]. Due to the absence of effective treatments beyond Riluzole, which at best extends lifespan by a few months, there is an urgent demand for novel therapeutic strategies for ALS patients [152]. Mutations in genes encoding the MAM proteins VAPB and Sig‐1R are implicated in ALS pathogenesis [153]. Additionally, mutations in key ALS‐related genes, including TAR DNA binding‐protein 43 (TDP‐43), fused in sarcoma (FUS), superoxide dismutase 1 (SOD1), and the C9orf72 repeat expansion, have been associated with impaired mitochondrial function and Ca^2+^ regulation [154]. Interestingly, these genes do not code for mitochondrial or ER proteins [154].

Mutations in VAPB can lead to ALS, exhibiting a wide range of clinical manifestations [96]. These include severe ALS with rapid disease progression, autosomal‐dominant ALS8, and late‐onset spinal muscular atrophy in affected individuals [96]. When VAPB undergoes mutation, it forms inclusions within the ER, leading to structural alterations in the ER [96]. The VAPB P56S mutation, a well‐known cause of familial ALS type‐8, has been shown to accumulate and exacerbate MERCS [131]. This mutation affects the interaction between the MAM protein VAPB and PTPIP51, leading to the formation of the MAM tether complex, which ultimately disrupts Ca^2+^ homeostasis [131, 155]. In neurons carrying mutant VAPB, dysregulation of Ca^2+^ within the ER leads to decreased mitochondrial transport [156]. This could be due to the influence of Ca^2+^ on Miro1, a cargo adaptor that bridges mitochondria to kinesins and microtubules [156]. Since mutant VAPB can trap the wild‐type VAPB protein, this interaction may reduce VAPB levels [157]. This could change the lipid composition of membranes and disrupt vesicle formation in ALS [157]. Additionally, phosphoinositide phosphatase Sac1, another VAP binding partner, plays a role in ALS8 pathogenesis [158]. In drosophila, the ortholog of VAPB, known as VAP33, has been found to interact with the oxysterol binding protein (OSBP) [152]. This interaction is crucial for the proper localization of OSBP in the ER [152]. However, ALS‐associated mutations in VAPB have been shown to disrupt this interaction with OSBP, preventing its correct localization in the ER [155]. Furthermore, the disruption of VAPB‐PTPIP51 tethers emerges as an initial characteristic in mutant C9orf72 transgenic mice, preceding the manifestation of disease symptoms [159]. Given the significance attributed to early pathogenic changes, this discovery underscores the critical role of VAPB‐PTPIP51 tether disruption in the pathogenesis of ALS [159].

Genetic mutations in the coding sequence of Sig‐1R have also been implicated in the development of ALS [153]. The Sig‐1R, an ER protein, serves as a chaperone for IP3R, thereby facilitating the delivery of Ca^2+^ to mitochondria [160]. Mutations in Sig‐1R lead to ALS because the mutant protein is unable to bind to IP3R3, resulting in a detachment of the ER from mitochondria [161]. This dissociation disrupts calcium homeostasis and impairs ATP synthesis, subsequently stalling axon extension, ultimately leading to the development of ALS in animals [161]. A homozygous E102Q mutation within a conserved transmembrane segment of Sig‐1R has been identified as the underlying cause of a juvenile autosomal recessive form of ALS16 [153]. In SOD1 mutant mice, the destruction of Sig‐1R expedited the onset of disease, concurrent with the dismantling of the MAM structure [105]. A reduction in Sig‐1R function may contribute to ALS pathology by inducing aberrant ER morphology, destabilizing lipid rafts, disrupting calcium signaling, and compromising ER‐Golgi trafficking [152]. The proximity of Sig‐1R to the muscarinic acetylcholine receptor at the plasma membrane hints at a potential role in ALS pathogenesis [162]. Furthermore, the interaction between Sig‐1R and Insig may contribute to the disease etiology, given that Insig has been implicated in reducing glutamate‐induced excitotoxicity, a key mechanism in ALS [163]. Therapy using Sig‐1R agonists has proven effective in various ALS models [131]. By targeting Sig‐1R with agonists, it may be possible to mitigate protein aggregation and slow disease progression because of the agonists' ability to enhance the chaperone activity of Sig‐1R [164].

TDP‐43 accumulation is a notable feature in ALS pathology, and mutations in the TDP‐43 gene are found in about 3% of familial ALS cases and 1.5% of sporadic cases [165]. TDP‐43 is a versatile protein that binds to both RNA and DNA, playing a role in transcription, transportation, and translation processes of RNA [105]. The misfolding, mislocalization, and hyperphosphorylation of TDP‐43 represent characteristic pathological aspects of ALS [166]. TDP‐43 modifies MAM tethering and Ca^2+^ homeostasis through disrupting the interaction between VAPB and PTPIP51 [121]. Notably, this disruption is mediated via the activation of GSK‐3β [113]. Hence, GSK3β inhibitors could potentially offer therapeutic benefits for ALS [160]. Following IP3R‐mediated Ca^2+^ release from the ER, TDP‐43 elevation increases cytosolic Ca^2+^ levels while reducing mitochondrial Ca^2+^ levels [113]. This aligns with weaker ER–mitochondria connections [113]. These disruptions in cellular Ca^2+^ balance have extensive implications, affecting mitochondrial ATP synthesis, mitochondria transport, autophagy, and ER stress, all of which are variably impacted in ALS [98]. Moreover, the overexpression of wild‐type or ALS‐associated mutant forms of TDP‐43 leads to the misplacement of Miro1 into the cytosol [113].

FUS, another significant pathological aspect of ALS, has also been demonstrated to disrupt the association between VAPB and PTPIP51, as well as the tethering of MAMs, via the activation of GSK‐3β [167]. The mutated FUS gene in ALS interacts with the valocin‐containing protein (VCP), potentially elucidating certain mitochondrial abnormalities observed in the disease [168].

Over 150 mutations identified in the SOD1 gene account for approximately 20% of familial ALS occurrences [110]. These mutants interact with Bcl‐2, impacting calcium balance [110]. VDAC is recognized as a molecular target affected by damage from ALS mutant SOD1 [169]. Studies have shown that physical association with mutant SOD1 aggregates partially inactivates VDAC [169]. In addition, mutant SOD1 disrupts ER‐mitochondria signaling by impairing Sig‐1R function [161].

There is something else that causes ALS. Most familial ALS cases are caused by C9orf72 gene mutations [170]. Research indicates that in the spinal cord of ALS patients, there is an elevation in levels of sphingomyelin, ceramides, and cholesterol esters, likely due to oxidative stress [171]. Consequently, it is plausible to suggest that in the ALS spinal cord, heightened synthesis of cholesteryl ester from cholesterol by lecithin cholesterol esterase leads to reduced cholesterol levels at MAMs and augmented ER‐mitochondrial interactions [152]. Mutations in genes governing lipid droplet biogenesis and dynamics have been found to influence the ALS phenotype in various model organisms, including mice, drosophila, and worms [172]. The dysfunction of lipid droplet biogenesis may have a significant impact on the pathological aspects of human VAPB‐mediated ALS [173]. In a comprehensive genome‐wide study, polymorphism in the IP3R has been linked to a heightened susceptibility to ALS [174]. The specific accumulation of IP3R3 in motor neurons implies that the preservation of MAM integrity is pivotal for understanding the selective susceptibility observed in ALS [161].

Like Alzheimer's disease and Parkinson's disease, ALS urgently needs treatment strategies. The latest study reveals MAM defects in oligodendroglia from induced pluripotent stem cells, linked to abnormal lipid metabolism, ER stress, mitochondrial issues, and reduced Ca^2+^ signaling [175]. This discovery offers new insights into the role of mutant FUS in oligodendroglial function and motor neuron degeneration. Recent studies also suggested that the Sig‐1R–AAA ATPase domain‐containing protein 3A (ATAD3A) axis and MAM‐TANK‐binding kinase 1 (TBK1) axis could be a promising new therapeutic point for mitochondrial dysfunction in neurological disorders including ALS [176, 177].

### 4.4 Huntington's Disease

In 1872, Huntington's disease (HD) was first described by a 22‐year‐old American physician named George Huntington [178]. It is a rare and hereditary neurodegenerative disease characterized by the gradual emergence of motor dysfunction, psychiatric manifestations, and cognitive decline [179]. Globally, it is approximated that the occurrence of HD stands at 2.7 individuals per 100,000 [180].

Given the intricate and dynamic connections between the ER and mitochondria, proteins located at the MAMs play a pivotal role in the development of Huntington's disease [181]. HD is caused by an abnormal expansion of CAG repeats in the HTT gene [182]. This gene encodes a polyglutamine (polyQ) stretch located at the N‐terminal of the huntingtin protein (HTT), which is subsequently referred to as mutant HTT (mHTT) [182]. Evidence has accumulated indicating that the soluble N‐terminal of mHTT interacts with both the outer and inner mitochondrial membranes [182]. Notably, it directly interacts with the translocator of the inner mitochondrial membrane (TIM23), disrupting mitochondrial protein import [182]. Research has demonstrated that mHTT binds to IP3R, leading to increased sensitivity of IP3R to activation by IP3 in both planar lipid bilayers and primary medium spiny neurons [98]. These neurons are the most significantly affected in Huntington's disease [98]. Research indicates that poly Q‐induced toxicity in Huntington's disease is linked to Sig‐1R expression, which regulates NF‐κB‐mediated upregulation of antioxidants and reduction of ROS levels [181]. Furthermore, Hyrskyluoto et al. noticed that the administration of the Sig‐1R agonist, PRE084, not only enhanced cell survival but also assisted in mitigating the harmful consequences induced by PolyQ repeats [181]. Sig‐1R accumulates in neuronal inclusions containing mHTT, potentially boosting proteasome activity and facilitating mHTT degradation [183]. Additionally, the activation of NMDARs or increased activity of ER Ca^2+^ release channels (InsP3R, RyR) quickly triggers Ca^2+^ uptake into mitochondria [182]. Given the numerous intersections between HD pathology and PGC‐1α dysfunction, it is intriguing to hypothesize that PGC‐1α at the ER‐mitochondria interface holds a crucial significance [98]. The HTT, which carries a polyglutamine repeat mutation linked to HD, has been identified as a protein associated with ER [184]. Multiple models of HD have shown that VCP specifically relocates to mitochondria, where it interacts with mHTT, leading to excessive mitophagy and subsequent neuronal death [73]. The accumulation of polyglutamine‐expanded HTT within protein aggregates triggers ER stress, leading to the upregulation of BiP/GRP78, C/EBP homologous protein (CHOP), PDI, and Sig‐1R protein levels [185]. Additionally, mutant HTT's direct interaction with GTPase Drp1 boosts its enzymatic activity, causing mitochondrial movement defects and synaptic insufficiencies [181, 186]. In the postmortem striatum of HD patients, late‐stage HD exhibited smaller mitochondria with increased DRP1 protein levels and decreased expression of the fusion protein MFN1 [187]. In cardiac models of Huntington's disease expressing PolyQ77, Drp1 interacts with Fis1, resulting in excessive mitochondrial fragmentation, ROS production, and lysosomal dysfunction [188].

For treatment, a recent study has shown that morin hydrate can mitigate HD‐related ER stress, MERC, mitophagy, and apoptosis by inhibiting the mTOR/IRE1‐α signaling, IP3R/VDAC interaction, PINK1/ubiquitin/Mfn2 pathway, and cytochrome c/caspase 3 axis [189]. More treatments for HD should be further studied.

### 4.5 Traumatic Brain Injury

A traumatic brain injury (TBI) refers to a non‐congenital injury sustained by the brain, caused by an external physical force, potentially leading to temporary or irreversible impairment in functionality [190]. Worldwide, an estimated 70 million individuals suffer from traumatic brain injuries annually [191]. TBI stands out as a prominent cause of fatalities among younger adults, significantly contributing to mortality rates and disability globally [192].

TBI is primarily classified into two categories: primary and secondary brain injury [9]. The latter is intricately linked to various biochemical pathways, primarily cerebral inflammation and apoptosis, driven by factors such as mitochondrial dysfunction, autophagy, Ca^2+^ imbalance, oxidative stress, excitotoxicity, and free radical generation [9]. Notably, these processes have a close association with MAMs [9]. The experiment also demonstrated the increase of MAM functions in a mouse model of TBI [193]. Sig‐1R plays an important role in TBI. Research indicates that in brain tissues of both TBI patients and TBI mice, neural expression of Sig‐1R was elevated [194]. Upon activation following TBI, Sig‐1R receptors demonstrate the capacity to decrease lesion size, alleviate brain edema, and mitigate neurological impairments [195]. Moreover, neuroinflammation, primarily driven by activated microglia and astrocytes, is a well‐recognized marker of secondary brain injury following TBI and is a significant contributor to chronic brain damage post‐TBI [192]. Prior research has shown that Sig‐1R is widely expressed in microglia and astrocytes, regulating the neuroinflammatory processes mediated by these glial cells in various central nervous system disorders [192].

Many other organizations in the MAM also play a role in the TBI. Studies revealed that autophagy functions as a suppressor of NLRP3 inflammasome activation [196]. Activation of the NLRP3 inflammasome can trigger the processing and release of IL‐1β and IL‐18, thereby intensifying the inflammatory response following TBI [197]. The expression of GRP‐78 was notably elevated in brain samples from both TBI patients and TBI mice, suggesting the presence of ER stress in the brain following TBI [194]. High levels of cytosolic Ca^2+^ can break down cytoskeletal and extracellular matrix proteins [198]. This increases ROS production, which plays a major role in the development of TBI [198, 199]. Research indicates that PERK, a MAM component crucial in regulating ER‐mitochondria interactions and mitochondrial apoptosis after TBI, modulates ROS signaling between the ER and mitochondria [81]. Moreover, nuclear factor‐erythroid 2‐related factor 2 (Nrf2), an anti‐oxidative stress factor and direct substrate of PERK, can be influenced by MAMs, thus protecting against TBI through its regulation of microglial function [9].

Recent studies have shown that activating Sig‐1R may offer a promising clinical strategy for pharmacological treatment in TBI patients [192, 194]. More treatments for TBI should be further studied.

### 4.6 Glaucoma

Glaucoma constitutes a set of conditions that cause irreversible vision loss, notably marked by the progressive deterioration of retinal ganglion cells [200]. Globally, it is a significant factor contributing to irreversible blindness [201]. An increase in intraocular pressure and relative hypoxia in the retina trigger the generation of ROS, subsequently placing the retina and optic nerve under chronic stress [202].

In response to hypoxic conditions stemming from inadequate oxygen transport, glaucoma‐related axonal transport deficiencies arise [203]. Under these conditions, FUNDC1 undergoes a significant increase in MAMs, recruiting DRP1 to enhance mitochondrial fission, ultimately initiating mitophagy [203]. This mitophagy triggers the involvement of another key player, the NLRP3 inflammasome (the only inflammasome associated with MAM dysregulation) [203, 204]. While NLRP3 is typically found in the ER, when activated by mitophagy/autophagy or ROS, it migrates from the ER to MAMs and connects with the adaptor protein ASC, initiating the assembly of the NLRP3 inflammasome [203]. In a glaucoma rat model involving ocular hypertension, a notable upregulation of Grp78 and CHOP, two proteins within the PERK signaling pathway, was observed [205]. Furthermore, blocking the PERK‐eIF2‐CHOP pathway exhibited protective effects on retinal ganglion cell soma and axons across various mouse glaucoma models [205, 206]. Chronic ocular hypertension elevates the levels of cleaved‐poly ADP‐ribose polymerase (PARP) and NLRP3, leading to disturbances in MAM regulation and mitochondrial malfunction [204]. Moreover, it has been shown that when retinal ganglion cells undergo apoptosis due to hydrostatic pressure, there is an increase in calcium levels within these cells [205].

At present, there is a lack of research on the treatment of glaucoma through MAMs, and we can further study this in the future to find new targets for the treatment of glaucoma.

### 4.7 Charcot–Marie–Tooth Disease

Charcot–Marie‐Tooth (CMT) disease is a genetically diverse group of inherited neuropathies [207]. It is typically marked by both sensory and motor problems and is often called hereditary sensory and motor neuropathy [207]. Typically, patients display distal muscle weakness and atrophy, accompanied by reduced ankle dorsiflexion, diminished tendon reflexes, and prominent foot arches (known as Pes cavus deformities) [208]. Additionally, they often experience a slight to moderate loss of distal sensation, predominantly occurring in a symmetric stocking–glove distribution, which often parallels the presentation of muscle weakness [209].

The etiology of CMT disease lies in abnormalities within axons, myelin sheaths, or both [210]. Among the axonal variants of CMT, a specific subtype known as CMT2A is attributed to mutations in the MFN2 gene [211]. Research indicates that a disrupted interplay between the ER and mitochondria plays a contributory role in the development of axonopathy observed in CMT2A transgenic mice [210]. In contrast, multiple studies have documented that mutations in the MFN2 gene associated with CMT2A disrupt the contact points between the ER and mitochondria, resulting in impairments in mitochondrial dynamics and their distribution within axons [96]. Additionally, reducing the activity of GDAP1, a protein linked to CMT when mutated, weakens the interaction between the ER and mitochondria [96]. This also disrupts Ca^2+^ balance in both vitro and vivo models [96]. GDAP1 is an outer mitochondrial membrane protein [100]. The absence of GDAP1 triggers alterations in mitochondrial networks and their interactions with ER, causing reduced ER‐Ca^2+^ levels and impairments in store‐operated calcium entry [100]. This dysfunction is attributed to a mislocalization of mitochondria to subplasmalemmal sites [100]. Motor neurons derived from GDAP1^−/−^ mice exhibited a decrease in the quantity of ER‐mitochondria interfaces, leading to diminished ATP production and disturbances in the axonal transport of mitochondria [212]. Moreover, the gene responsible for encoding IP3R3 has been proved to be a contributor to CMT, and fibroblasts from affected patients exhibited imbalances in Ca^2+^ homeostasis [213].

The latest study has corrected the root genetic defect of CMT2A in a mouse model, and future research should continue into the possibility of application in humans [214].

### 4.8 Wolfram Syndrome

Wolfram syndrome is distinctively marked by the early onset of diabetes mellitus, accompanied by diabetes insipidus, optic nerve deterioration, auditory impairment, and progressive neurodegeneration [215]. It is a rare autosomal recessive condition, occurring in approximately one out of 700,000 individuals [216]. Many patients have harmful mutations in the WFS1 gene [217]. Besides, a recessive mutation in the CISD2 gene encoding CDGSH iron–sulfur domain‐containing protein 2 has been reported in rare cases of type 2 Wolfram syndrome [217].

A portion of this disease stems from mutations in the WSF1 gene, which encodes the wolframin protein, a transmembrane protein of the ER that plays a role in MAMs [218]. An initial electrophysiological investigation of Xenopus oocytes showed an interaction between WFS1 and IP3Rs [219]. By examining channel activity in lipid bilayers, it was observed that the incorporation of WFS1 into these bilayers resulted in an augmented IP3‐triggered current [219]. Conversely, neurons lacking WFS1 exhibited reduced IP3R‐mediated Ca^2+^ release and disrupted mitochondrial dynamics, thereby contributing to delayed development [220]. At the MAMs, WFS1 interacts with Sig‐1R, facilitating the positive regulation of calcium transfer between the ER and mitochondria [219]. Research has shown that the activation of Sig‐1R has the potential to mitigate the functional impairments in MAMs resulting from wolframin deficiency [218]. Utilizing PRE‐084, an agonist of the Sig‐1R, to stimulate the receptor can effectively counteract the WFS1 deficiency‐induced decrement in IP3R‐mediated Ca^2+^ release and subsequent ER‐mitochondrial Ca^2+^ transfer [218]. Moreover, VDAC1 has been determined to interact with WFS1, and the loss of this interaction in WS cells may negatively impact mitochondrial function [221]. However, replenishing WFS1 levels in WS cells reinstates the WFS1‐VDAC1 interaction, which is associated with an expansion in MAMs and mitochondrial networks, potentially enhancing mitochondrial function [221].

WS type 2 is a condition that arises from mutations in the CISD2 gene, encoding a protein primarily expressed in the MAMs [105]. According to transmission electron microscopy findings, fibroblasts from WS type 2 patients exhibit a notable increase in ER‐mitochondrial contacts compared to healthy controls [222]. CISD2 holds a pivotal position in regulating cytosolic Ca^2+^ homeostasis as well as maintaining ER integrity and mitochondrial functionality [223]. Rouzier et al. provided experimental evidence suggesting that imbalances in Ca^2+^ homeostasis and disrupted ER‐mitochondrial interactions are significant pathophysiological factors that ultimately contribute to the development and progression of CISD2‐related diseases [217]. Cisd2, IP3R, and Bcl‐2 constitute a macrocomplex, essential for regulating Ca^2+^ signaling and the physiology of MAMs [224]. Biophysical investigations have confirmed that the Cisd2‐Bcl‐2 complex functions through the interaction between Cisd2's catalytic domain and Bcl‐2's BH4 domain, which is also responsible for inhibiting IP3R [219].

Recent studies demonstrated that ER calcium depletion plays a crucial role in disrupting ER‐to‐mitochondria calcium transfer and causing mitochondrial dysfunction in Wolfram syndrome [225]. This presents a significant opportunity for treating neuronal diseases linked to defective ER‐mitochondria contact sites, including Wolfram syndrome. Another recent study suggests that future research should focus on neuron–glia communication to gain a deeper understanding of WS pathogenesis [226].

## 5 Conclusions

In summary, MAMs serve as a key node for the interaction between mitochondria and ER, and their dysfunction is a common feature in multiple neurodegenerative diseases. Through this review, we delve into the significance of MAMs as a crucial platform for the communication between mitochondria and the ER in regulating intracellular signaling and metabolism. For numerous neurodegenerative diseases, including Alzheimer's disease, Parkinson's disease, and amyotrophic lateral sclerosis, we provided a detailed analysis of how MAM dysfunction contributes to the onset and progression of these diseases. Specifically, MAMs exhibit significant impacts on regulating APP processing, α‐synuclein aggregation, mitochondrial ATP production, and mitochondrial dynamics, among other changes that subsequently trigger neuronal dysfunction and death. Furthermore, this review highlights the critical roles of the Sig‐1R and other MAM‐associated proteins in maintaining ER‐mitochondrial communication, closely linking their dysfunction to various neurodegenerative diseases.

Through the analysis of the effects of MAM on neurodegenerative diseases, we found that the relationship between MAM and neurodegenerative diseases is not well understood. We found something that was contradictory in previous studies and worthy of further investigation, such as in Parkinson's disease, α‐synuclein overexpression or mutation disrupts Ca^2+^ homeostasis, leading to increased ATP levels. At the same time, α‐synuclein interacts with VAPB, destroying VAPB‐PTPIP51 and resulting in decreased ATP levels. Also in Parkinson's disease, the interaction between PINK1 and Beclin1 leads to increased mitochondrial contact in the ER, which in turn promotes the formation of autophagosomes. In the process of autophagy, MFN2 ubiquitination increases, leading to the dismantling of MFN2 chains, which in turn leads to reduced mitochondrial contact in the ER. Similarly, α‐synuclein mutations cause Parkinson's disease and lead to decreased ER mitochondrial contact. PINK and Parkin mutations cause Parkinson's disease but lead to increased ER mitochondrial contact. Recent research suggests that communication between ER and mitochondria may play a double‐edged role. This partly explains these contradictions. However, the current research is not comprehensive. Among these conditions, it is crucial to investigate the tissue‐specific function of MAMs and delve into methods to precisely regulate their formation and function. The latest research findings have shown that ERMCSs possess unique subdomains and exhibit highly dynamic characteristics, where tethers diffuse in and out of the site within milliseconds, and the size and configuration of contact sites vary in response to various stimuli. We should do more in‐depth research based on this in the future. In addition, for Alzheimer's disease and other neurodegenerative diseases, the molecular mechanism of MAMs has not been fully understood, but we can analyze the existing literature to show that key proteins such as Sig‐1R, IP3R, and VAPB play an important role in most neurodegenerative diseases. Ca^2+^ also plays an unparalleled role. Lipids such as cholesterol also participate in many neurodegenerative diseases. These may offer new ideas and approaches for the treatment of neurodegenerative diseases. In the future, we can develop more effective targeted therapeutic drugs for these proven key substances.

## Author Contributions

The review was designed by Y.L., X.G., and Y.Z. Related articles were screened and analyzed by Y.Z. The manuscript of this review was prepared by Y.Z. Y.L., X.G., X.W., F.H., and X.R. critically revised the draft before submission. J.W., H.L., and Q.W. helped perform the analysis with constructive discussions. All authors contributed to the article. All authors read and approved the final version.

## Conflicts of Interest

The authors declare no conflicts of interest.

## Data Availability

The authors have nothing to report.
